# Ascending noradrenergic excitation from the locus coeruleus to the anterior cingulate cortex

**DOI:** 10.1186/s13041-020-00586-5

**Published:** 2020-03-26

**Authors:** Kohei Koga, Akihiro Yamada, Qian Song, Xu-Hui Li, Qi-Yu Chen, Ren-Hao Liu, Jun Ge, Cheng Zhan, Hidemasa Furue, Min Zhuo, Tao Chen

**Affiliations:** 1 Center for Neuron and Disease, Frontier Institute of Science and Technology, Xi’an Jiaotong University, Xi’an, 710049 China; 2 Department of Physiology, Faculty of Medicine, University of Toronto, Medical Science Building, 1 King’s College Circle, Toronto, Ontario M5S 1A8 Canada; 3 Department of Neurophysiology, Hyogo College of Medicine, Nishinomiya, 663-8501 Japan; 4 Department of Anatomy, Histology & Embryology, Air Force Medical University, Xi’an, 710032 China; 5 National Institute of Biological Sciences, Beijing, 102206 China; 6 Tsinghua Institute of Multidisciplinary Biomedical Research, Tsinghua University, Beijing, 102206 China

**Keywords:** Noradrenaline, Anterior cingulate cortex, Glutamatergic transmission, Pyramidal neuron, Layer II/III, Optogenetics

## Abstract

Anterior cingulate cortex (ACC) plays important roles in sensory perception including pain and itch. Neurons in the ACC receive various neuromodulatory inputs from subcortical structures, including locus coeruleus noradrenaline (LC-NA) neurons. Few studies have been reported about synaptic and behavioral functions of LC-NA projections to the ACC. Using viral-genetic method (AAV-DIO-eYFP) on DBH-cre mice, we found that LC-NA formed synaptic connections to ACC pyramidal cells but not interneurons. This is further supported by the electron microscopic study showing NAergic fibers contact the presynaptic inputs and post-synaptic areas of the pyramidal cells. NA application produced both pre- and post-synaptic potentiation effects in ACC excitatory transmission in vivo and in vitro. Activation of LC-NA projection to the ACC by optogenetic method produced enhancement of excitatory transmission in vitro and induced scratching and behavioral sensitization for mechanical stimulation. Our results demonstrate that LC-NA projections enhance or facilitate brain responses to pain and itch by potentiating glutamatergic synaptic transmissions in the ACC.

## Introduction

The locus coeruleus (LC) is a main origin of noradrenergic neurons [1–3]. Noradrenaline (NA) is a key neuromodulator to play critical roles in various higher brain functions in the central nervous system (CNS) [1, 2]. In the brain, NA contributes to arousal, attention, cognition and memory [4, 5]. NA also plays roles as modulatory systems for pain sensation in the spinal dorsal horn [6–8].

Sensory transmission is under the control of endogenous modulatory systems [6, 9–12]. Spinal cord plays a crucial role in the transmission and modulation of painful information [13, 14]. It has been reported that spinal noxious sensory transmission can be modulated by descending inhibitory modulation [6, 11] and/or facilitatory modulation [12, 15, 16]. The LC is a key structure which contributes to descending modulation of painful information in the spinal cord [17–19]. The activation of LC triggers descending inhibition to the spinal cord, and produces analgesic effect by releasing NA in the spinal cord [6].

In addition to the well-known descending projection, LC sends projections to supraspinal structures including cortical areas [20, 21]. Cortical areas, including the anterior cingulate cortex (ACC) and the insular cortex (IC), play important roles in chronic pain and emotional responses [20, 22–25]. Selective activations of the ACC and/or IC by electric stimulations or optogenetic stimulation induce modulatory effects on nociception and emotion in monkey and rodents study [20, 26, 27]. Electrical stimulation or optogenetic selective activation of pyramidal neurons acutely reduces nociceptive thresholds [26, 27]. In contrast, inhibition of pyramidal neurons in the ACC reduces hypersensitivity induced in chronic inflammatory pain model [26]. A similar analgesic effect was induced by the activation of parvalbumin-expressing interneurons in the ACC [26]. These results in animals are consistent with human imaging studies that demonstrate that the ACC is a critical area for chronic pain [20, 22, 24]. In addition to pain perception, human imaging study show that the ACC is a critical cortical area for itch sensation [28, 29]. Rodent studies demonstrate that itch stimulation can activate neurons in the ACC [30, 31].

Although descending NAergic LC-spinal system has been documented [4, 5], the functions of NAergic LC-ACC projections are not investigated yet. It is unclear if such NAergic projection may also produce analgesic effects as their descending modulation to the spinal dorsal horn. In the present study, we take advance of the optogenetic approach to investigate the selective effect of LC-ACC projection on synaptic transmissions within the ACC as well as behavioral responses. We found that ascending LC-ACC projections enhance glutamatergic synaptic transmission and neural excitability in the ACC, and facilitated behavioral responses to pain and itch sensory stimulation in mice. Different with the well-known mechanism of NA in the spinal cord level, our work for the first time reveals the mechanism of NA in the LC-cortical ascending pathway in sensory modulation.

## Materials and methods

### Animals

Adult male C57BL/6 and DBH-cre mice (8–12 week old) were used. Male Sprague-Dawley rats (SD rats) were used for in vivo extracellular recording. Adenylyl cyclase type 1 (AC1) knockout (KO) and AC8 KO mice were a gift from Dr. Daniel R. Storm (University of Washington, Seattle, WA) [32–34] and were maintained on a C57BL/6 background. All mice and rats were maintained on a 12-hlight/dark cycle (temperature 22–26 °C, air humidity 55–60%) with food and water provided ad libitum. The Animal Care and Use Committee at the Air Force Medical University and Xian Jiaotong University in China, National Institutes of Natural Sciences in Japan and Hyogo College of Medicine approved the experimental protocols.

### In vitro whole-cell patch-clamp recordings in the ACC slices

Experimental procedures were based on those described previously [35–37]. Briefly, mice were anesthetized with 1–2% isoflurane and coronal brain slices including the ACC (300 μm) were prepared using standard methods. Slices were transferred to a room temperature-submerged recovery chamber with an oxygenated (95% O_2_–5% CO_2_) artificial cerebrospinal fluid (ACSF) containing (in mM) 124 NaCl, 25 NaHCO_3_, 2.5 KCl, 1 KH_2_PO_4_, 2 CaCl_2_, 2 MgSO_4_ and 10 glucose. After a 1 h recovery period, slices were transferred into a recording chamber on the stage of an Axioskop 2FS microscope (Zeiss) equipped with infrared DIC optics for visualized recordings. All experiments were recorded with an Axon 200B amplifier (Axon Instruments). In the voltage-clamp configuration, recording electrodes (2–5 MΩ) contained the pipette solution composed of (in mM) 120 K-gluconate, 5 NaCl, 1 MgCl_2_ 0.5 EGTA, 2 Mg-ATP, 0.1 Na_3_GTP, and 10 HEPES; pH 7.2, 280–300 mOsm. The membrane potential was held at − 60 mV for recording spontaneous excitatory post-synaptic currents (sEPSCs) and held at 0 mV for recording spontaneous inhibitory post-synaptic currents (sIPSCs). APV (50 μM) was always added in the ACSF. Picrotoxin (100 μM) was added into the ACSF for recording sEPSCs and CNQX (25 μM) was added into the ACSF for recording sIPSCs. The pipette solution was containing (in mM): Cs-MeSO_3_, 120; NaCl, 5; MgCl_2_ 1; EGTA, 0.5; Mg-ATP, 2; Na_3_GTP, 0.1; HEPES, 10; pH 7.2; 280–300 mOsmol. Pyramidal neurons and interneurons were distinguished based on their morphology, membrane properties and firing pattern (Fig. 6a). The initial access resistance was 15–30 MΩ, and it was monitored throughout the experiment. Data were discarded if the access resistance changed > 15% during experiment. Data were filtered at 1 kHz, and digitized at 10 kHz. Data were collected and analyzed with Clampex and Clampfit 10.2 software (Axon Instruments). NA and NA receptors agonist or antagonists were bath applied in ACSF.

In some cases, biocytin (0.5%) were added into the pipette solution for labeling the morphology of the recorded pyramidal cells or interneurons. After recording, the slices were fixed in 4% paraformaldehyde in 0.1 M phosphate buffer (PB, pH 7.4) for 1 h at room temperature. Slices were then rinsed with 3% hydrogen peroxide in PBS for 30 min and throughly washed with 0.01 M PBS (pH 7.4). The tissue was then incubated with Alex594 conjugated Streptavidin (1:200, Jackson) for 4 h at room temperature. The immunofluorescence-labeled sections were then rinsed in PBS, mounted onto glass slides and visualized under confocal microscope under appropriate filter.

### In vivo electrophysiology for ACC recording

In vivo preparations were made as described previously [38]. Under urethane anesthesia (1.2–1.5 g/kg, i.p.), rats were mechanically ventilated after tracheostomy and bilateral thoracotomy was performed. After the head was fixed in a stereotaxic apparatus (Model SR-6R, Narishige, Tokyo, Japan), a craniotomy is performed using a dental drill to open a hole above the ACC according to the stereotaxic coordinates. A tungsten electrode (impedance, 10 MΩ, A-M systems, Sequim, WA) was placed into the ACC, and conventional extracellular recordings were obtained as shown previously [39] with an AC differential amplifier (DAM 80, World Precision Instruments, Sarasota, FL). Data were filtered (300 to 5 kHz) and digitized (10 kHz). Unit firings were sorted with Offline Sorter software (version 3, Plexon, Dallas, TX). Putative pyramidal neurons in the ACC were identified based on their waveforms as shown previously [40]. In the case of drug microinjection into the ACC, a cannula was inserted through the same hole, and the tip was placed in the vicinity (approximately 1 mm) of the recording electrode. Drugs (noradrenaline, 50 μg/0.5 μL; phenylephrine, 5 μg/0.5 μL; Isoproterenol, 37 μg/0.5μL) diluted in normal saline were microinjected over a 5 min period. For the LC electrical stimulation, a hole was further opened on the skull above the cerebellum ipsilateral to the recording site, and a combined bipolar stimulating electrode-tungsten extracellular recording electrode (1 MΩ, A-M systems, Sequim, WA) was stereotaxically inserted into the floor of the fourth ventricle above the LC. The electrode was then lowered and placed into the LC if neuronal activity is considered to be obtained from LC neurons. LC neurons were identified based on their characteristic spontaneous firing and responses to contralateral cutaneous noxious stimulation as described previously [41, 42]. Trains of electrical pulses (duration 200 μs, 50 times, 4 ms interval) were then applied with an interval of 2 s for 1–2 min.

### Immunohistochemistry

Immunostaining procedure was applied as described previously [15]. In brief, mice were perfused with 0.1 M PBS and 4% paraformaldehyde. After fixation, brainstem containing LC and brain containing ACC were removed, cryoprotected and serially cut into transverse slices with 30 μm thickness. The sections were then rinsed in PBS with 0.3% Triton X-100 and 1% normal goat serum (NGS) for 0.5 h. LC sections were dual-immunostained with GFP and TH. Sections were incubated with mouse anti-GFP antibodies (1:500, Abcam, ab183734) and rabbit anti-TH (1:500, Abcam, ab112) for 24 h, following with Alex594 conjugated anti-rabbit (1:1000, Jackson) and Alexa488 conjugated anti-mouse (1:1000, Jackson) antibodies overnight at room temperature. ACC sections were dual-immunostained for GFP/RFP or GFP/GAD. One set of series sections were incubated with mouse anti-GFP antibodies (1:500, Abcam) and rabbit anti-RFP antibody (1:500, Abcam, ab62341) for 24 h, following with Alex594 conjugated anti-rabbit (1:1000, Jackson) and Alexa488 conjugated anti-mouse (1:1000, Jackson) antibodies overnight at room temperature. Another set of series sections were incubated with mouse anti-GFP antibodies (1:500) and rabbit anti-GAD antibody (1:200, Abcam, ab203063) for 24 h, following with Alex594 conjugated anti-rabbit (1:1000) and Alexa488 conjugated anti-mouse (1:1000) antibodies overnight at room temperature. Sections were then rinsed in PBS, counterstained with DAPI antibodies for 5–10 min and mounted onto glass slides. The signals were visualized under confocal microscope under appropriate filter.

For the electron microscopic staining and observation, mice were deeply anesthetized and then transcardially perfused with normal saline, followed by 0.1 M PB containing 4% paraformaldehyde, 0.1% glutaraldehyde and 15% picric acid. Sections from the brain containing ACC were generated using a vibratome at a 50-μm thickness. Sections were then incubated overnight with mouse anti-GFP antibodies (1:200, Abcam) and rabbit anti-RFP antibody (1:200, Abcam), followed by anti-rabbit IgG conjugated to 5-nm gold particles (1:100, Nanoprobes) and biotinylated anti-mouse IgG (1:200, Abcam) for 4 h. Silver enhancement was performed with HQ Silver Kit (Nanoprobes) for visualization of RFP (mCherry) immunoreactivity. Then sections were incubated in the avidin-biotin peroxidase complex for 45 min and then incubated with diaminobenzidine (DAB) solution. Immunolabelled sections were then fixed with 0.5% osmium tetroxide in 0.1 M PB for 1 h, dehydrated in graded ethanol series and then in propylene oxide, and finally flat-embedded in Epon 812 between sheets of plastic. After polymerization, acrylic sheets were peeled from the polymerized resin, and flat-embedded sections were examined under the light microscope. Three to four sections containing GFP and RFP immunoreactivity were selected from each brain, trimmed under a stereomicroscope, and glued onto blank resin stubs. Serial ultrathin sections were cut with an Ultramicrotome using a diamond knife and mounted on formvar-coated mesh grids. They were then counterstained with uranyl acetate and lead citrate, and observed under a JEM-1230 electron microscope.

### Behavioural tests

Four to five weeks after AAV_2/9_- hEF1a-DIO-ChR2(H134R)-eYFP or AAV_2/9_-hEF1a - DIO-eYFP injection, mice were anesthetized with an intraperitoneal injection of ketaminexylazine (0.1 mg per gram body weight ketamine, 0.01 mg per gram body weight xylazine) and the head was fixed in a stereotaxic apparatus. A small craniotomy was performed and a hole were drilled. The optic cannula (MFC_200/230–0.39_ 2mm_ZF1.25_FLT, Doric Lenses., Quebec, Canada) was implanted in the middle line of ACC (0.98 mm anterior to Bregma and 1.0 mm deep from skull surface). The optic cannula was then fixed with dental cement [15].

One week after optic cannula implantation, mice with ChR2-eYFP or eYFP infection were placed into Lucite cubicles with a plain pedestal to observe the basal behavior. After acclimation for 5 min, optostimulation at 5 or 20 Hz (3 min) were applied through the implanted fibers by Master 8 automatically. The whole experiment process was video captured and analyzed by observers. The bouts and duration of scratching and wiping behaviors were collected and averaged per min before, during and after optostimulation. For testing the paw withdrawal thresholds, mice were places into Lucite cubicles over a wire mesh with von Frey filaments applied to their left and right hind paws. After acclimation for 5 min, series of filaments (0.008, 0.02, 0.04, 0.16, 0.4, 0.6, 1, 1.4, 2 g) with various bending forces (according to 0.078, 0.196, 0.392, 1.568, 3.92, 5.88, 9.8, 13.72, 19.6 mN) were manually applied to the plantar surface of the hindpaw until the mice withdrew from the stimulus. The lowest force at which a withdrawal response obtained was considered as the paw withdrawal threshold. The paw withdrawal thresholds before and after optostimulation (5 or 20 Hz, 3 min) were tested. In all experiments, observers were blind to animal grouping.

After behavior test, brains containing ACC were fixed and cut into transverse slices (30 μm). The sections were immunostained with rabbit-Fos antibodies (1:300, Abcam, ab190289), following with Alex594 conjugated anti-rabbit (1:1000, Jackson). ACC areas were then observed under confocal microscope for checking the Fos and LC-ACC projecting fiber expression.

### Optogenetics

AAV_2/9_- hEF1a-DIO-ChR2(H134R)-eYFP and AAV_2/9_-hEF1a-DIO-eYFP were purchased from Taitool Bioscience Co. Ltd. (ShangHai, China) and AAV_2/9_-CaMKIIα-mCherry was purchased from BrainVTA Co. Ltd. (WuHan, China). AAV_2/9_-DIO-ChR2-eYFP or AAV_2/9_-DIO-eYFP (1 × 10^13^, 200 nL per site) were injected into the dual sites of LC and AAV- CaMKIIα-mCherry (3 × 10^12^, 200 nL per site) were injected into the dual sites of the ACC in DBH-cre mice. After one month, the immunostaining, whole cell patch recording or in vivo behavioural experiments were carried out, respectively. For performing whole-cell patch recording or in vivo behavioural experiments, optostimulation was applied with 5 or 20 Hz blue light (470 nm, 5 ms pulse width in an intensity of 15 mW/mm^2^ at the optic fiber tip) [15, 26, 43, 44].

### Statistics

Statistical comparisons were made using the Student *t*-test, one-way or two-way ANOVA as appropriate. Significance between groups was tested with a Holm-Sidak or Tukey tests to adjust for multiple comparisons. All data were presented as the mean ± S.E.M. In all cases, *P* < 0.05 was considered statistically significant.

## Results

### Ascending projection from the LC to ACC

**Fig. 1:**
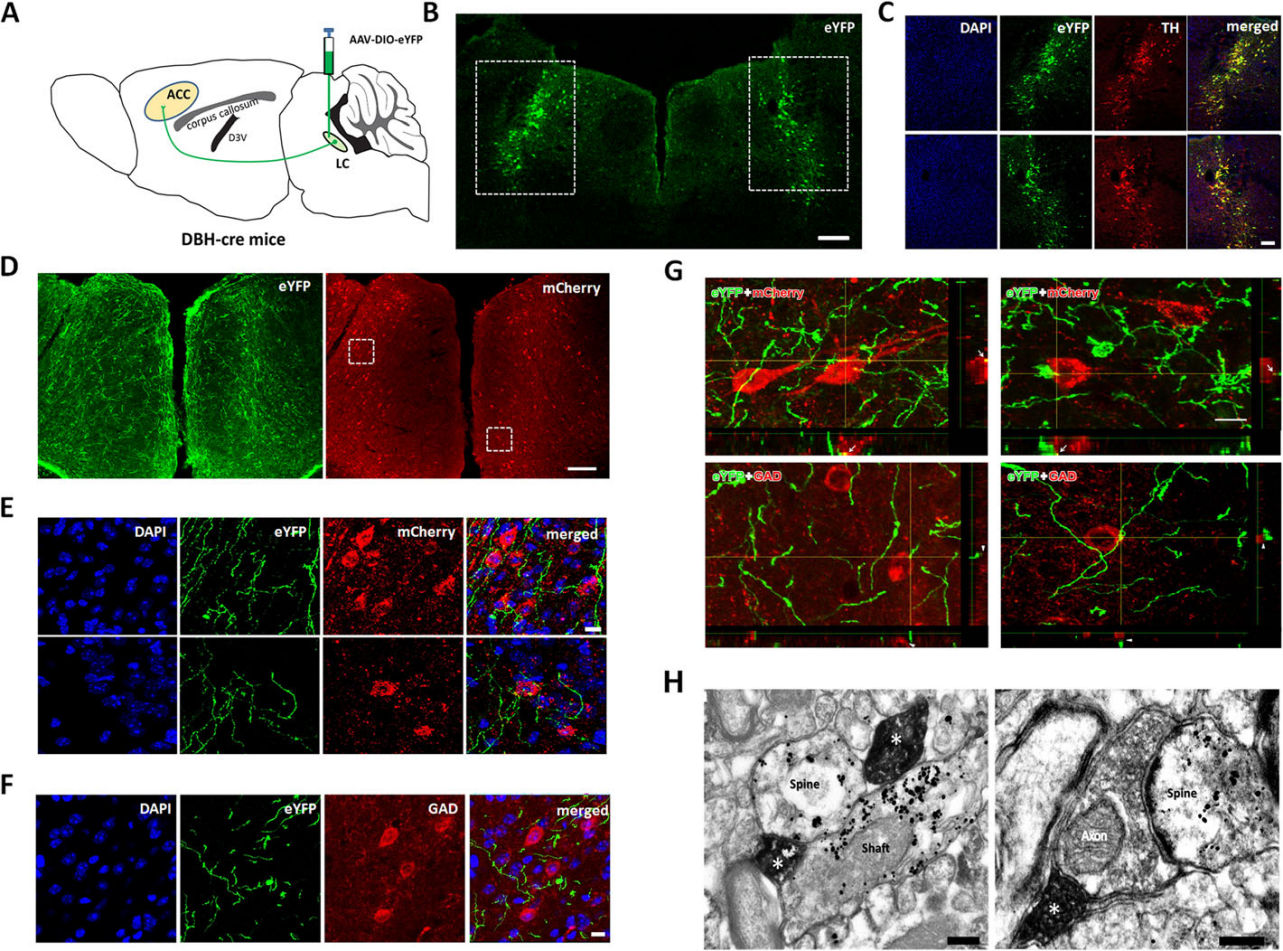
Noradrenergic fibers from the LC project to the postsynaptic pyramidal cells in the ACC. **a** Diagram of AAV-DIO-eYFP injection and anterograde tracing strategy in the DBH-cre mice. **b** The AAV-DIO-eYFP injection sites in the LC. **c** AAV infected neurons in the rectangle areas of **b** are dual labeled with TH- immunopositivities. **d** Distribution of LC-ACC projecting NAergic fibers showing eYFP and the pyramidal cells infected by AAV-CaMKIIα-mCherry in the ACC. **e** Enlarged figures in the rectangle areas in **d** showing LC-ACC projecting fibers (eYFP) made close connections with pyramidal cells (mcherry). **f** LC-ACC projecting NAergic fibers (eYFP) does not make close connection with GAD immunoreactive GABAergic neurons. **g** The 3D view of the close connections between eYFP labeled LC-ACC fibers and mcherry-labeled pyramidal cells or GAD-immunoreactive GABAergic neurons. **h** Two LC-ACC NAergic (eYFP immunoreactive with DAB) axon terminals (*) simultaneously make synapses with a dendritic spine and a shaft of a pyramidal cell (mcherry immunoreactive with nanogold) (left panel); One LC-ACC NAergic terminal (*) makes a synapse with a glutamatergic axon terminal, which in sequence makes a synapse with a spine of a pyramidal cell (right panel). Bars equals to 200 nm in **h**, 10 μm in **e**, **f** and **g**, 100 μm in ***C*** and 200 μm in **b** and **d**

We first used Cre-dependent virus tracing method to examine the NAergic projection from the LC to ACC by injecting AAV-DIO-eYFP into the LC in DBH-cre mice (Fig. 1a). Most of the NAergic neurons (96.4 ± 2.3% of TH immunoreactive (ir) neurons, *n* = 4 mice) in LC were successively infected (Fig. 1b-c) and their projecting fibers were distributed densely in both sites of the ACC (Fig. 1d). We labelled ACC pyramidal cells by injecting AAV-CaMKIIα-mCherry into both sites of ACC and found that LC-ACC projection fibers made close connections with mcherry-labeled pyramidal cells (Fig. 1d and e) but did not make close connections with GAD-ir GABAergic neurons (Fig. 1f). To further identify the connection between projecting fibers and pyramidal cells or interneurons, the three-dimensional view was introduced under the confocal microscope. We collected 242 RFP-labeling pyramidal cells (3 mice) and found that 198 (81.8%) of them were crossed or terminated by NAergic fibers/terminals. From three-dimensional view, it has indicated that the fibers/terminals made close connection with pyramidal cells (yellow dots indicated by arrows in Fig. 1g). However, in 102 GAD-immunostaining GABA neurons, we only found 12 (11.8%) of them were crossed by NAergic fibers/terminals. Further three-dimensional view showed that this kind of crossing is not likely to be close connection with, but just passing over the GAD-immunostaining neurons (see the places indicated by arrowheads in Fig. 1g).

Finally, we observed the synaptic connections between LC-ACC projecting fibers and ACC pydamidal cells under the electron microscopy (EM). The NA-immunireactive (ir) products were stained with DAB and distributed in axon terminals. Pyramidal cells were shown with nano-gold labelled mCherry distributed in the neuronal soma and dendritic spines and shafts. More than half of the identified pyramidal cells’ profiles were synapsed by NA-ir terminals (52.2% of 232 nano-gold containing profiles), indicating that NA have direct modulatory effect on the pyramidal cells (Fig. 1h left panel). NA-ir terminals synapsed with glutamatergic-like axon terminals were frequently observed. In many cases (*n* = 38), the contacted glutamatergic-like axon terminal also in turn made synapse with a pyramidal cell, suggesting that NA may regulatory the release of glutamate to the pyramidal cell (Fig. 1h right panel).

### Effects of NA on excitatory synaptic transmission in the ACC

**Fig. 2:**
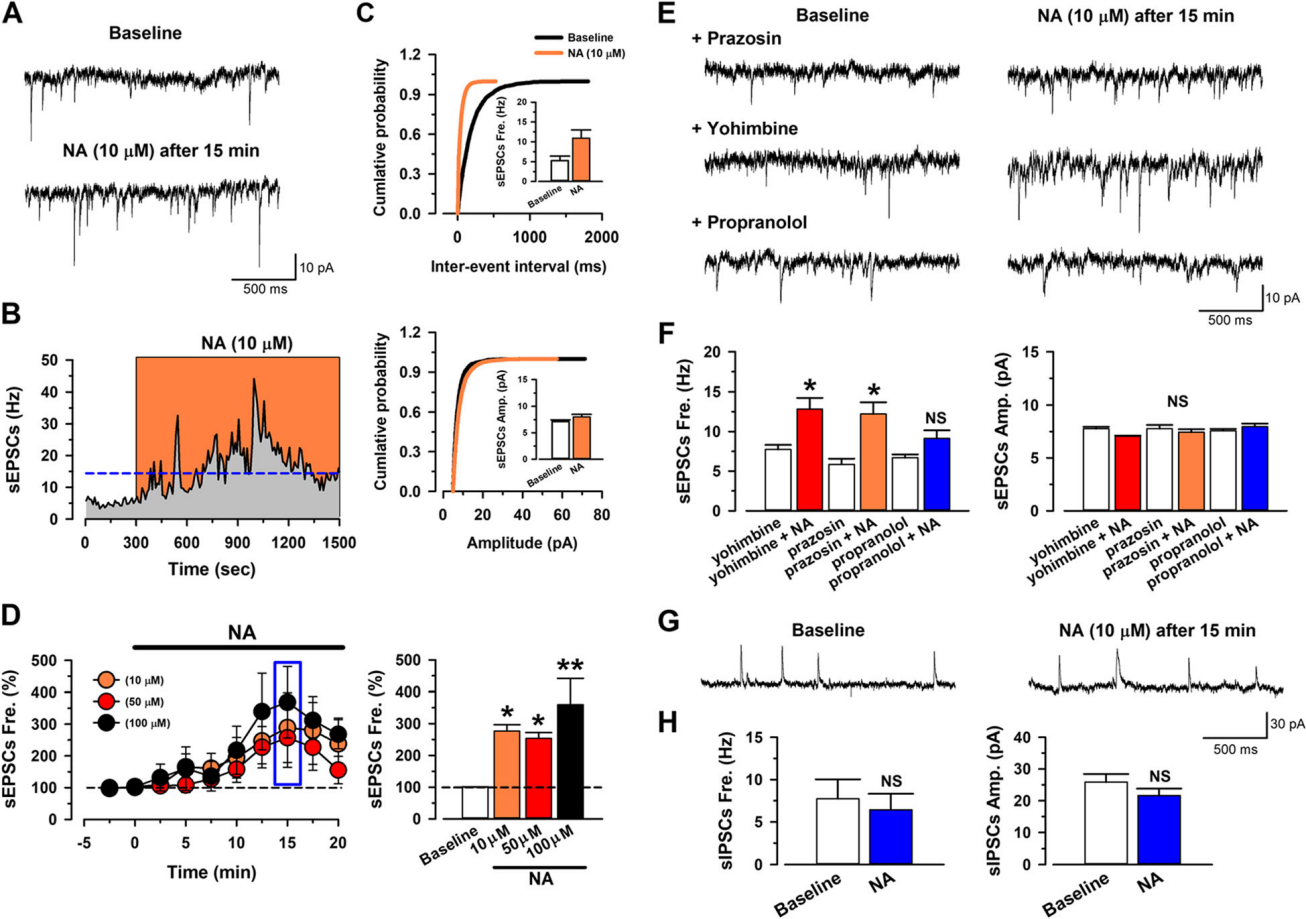
Noradrenaline facilitates the spontaneous glutamatergic transmitter release in layer II/III pyramidal neurons in the adult mice ACC via β receptor. **a** Raw trace of sEPSCs in baseline and NA (10 μM) after 15 min application. **b** The histogram showing the frequency of sEPSCs of neuron in **a**. **c** Cumulative probability histogram and averaged results showing the frequency and amplitude of sEPSCs before and after NA (10 μM) application. *, *p* < 0.05; ns, *p* > 0.05. *n* = 8, Paired *t*-test. **d** Averaged results showing the frequency of sEPSCs are increased after NA (10, 50 or 100 μM) application (10 μM: 277 ± 20% of baseline, *n* = 8; 50 μM: 253 ± 18% of baseline, n = 8; 100 μM: 359 ± 83% of baseline, *n* = 7). *P < 0.05, Baseline vs. NA (10 μM or 50 μM), **P < 0.01, Baseline vs. NA (100 μM), One-way ANOVA. **e** Sample traces of sEPSCs by NA (10 μM) after 15 min in the presence of an α1 receptor antagonist prazosin (1 μM), α2 receptor antagonist yohimbine (1 μM) or β receptor antagonist propranolol (1 μM). **f** The β receptor antagonist, but not α1 receptor antagonist nor α2 receptor antagonist blocks NA-induced enhanced frequency of sEPSCs (yohimbine: 7.75 ± 0.56 Hz; yohimbine + NA: 12.83 ± 1.40 Hz, *n* = 12; prazosin: 5.86 ± 0.71 Hz; prazosin + NA: 12.21 ± 1.48 Hz, *n* = 10; propranolol: 6.69 ± 0.42 Hz; propranolol + NA; 9.13 ± 1.03 Hz, n = 10). *P < 0.05, yohimbine vs. yohimibine + NA; prazosin vs. prazosin + NA (left panel). NS indicates no statistical significance between propranolol and propranolol + NA. Receptor antagonists do not change the averaged amplitude of sEPSCs (yohimbine: 7.77 ± 0.17 pA; yohimbine + NA: 7.06 ± 0.06 pA, n = 12; prazosin: 7.77 ± 0.35 pA; prazosin + NA: 7.44 ± 0.28 pA, n = 10; propranolol: 7.57 ± 0.17 pA; propranolol + NA; 7.96 ± 0.29 pA, n = 10) (right panel). Paired *t*-test. **g** Sample traces of sIPSCs in baseline (left) and NA (10 μM, right) after 15 min application. **h** Averaged frequency (Baseline: 7.73 ± 2.30 Hz; NA: 6.44 ± 1.88 Hz, *n* = 5; left panel) and amplitude (Baseline: 25.84 ± 2.56 pA; NA: 21.63 ± 2.18 pA, n = 5; right panel) of sIPSCs before and after NA application. NS indicates no statistical significance among the groups. Paired *t*-test

**Fig. 3:**
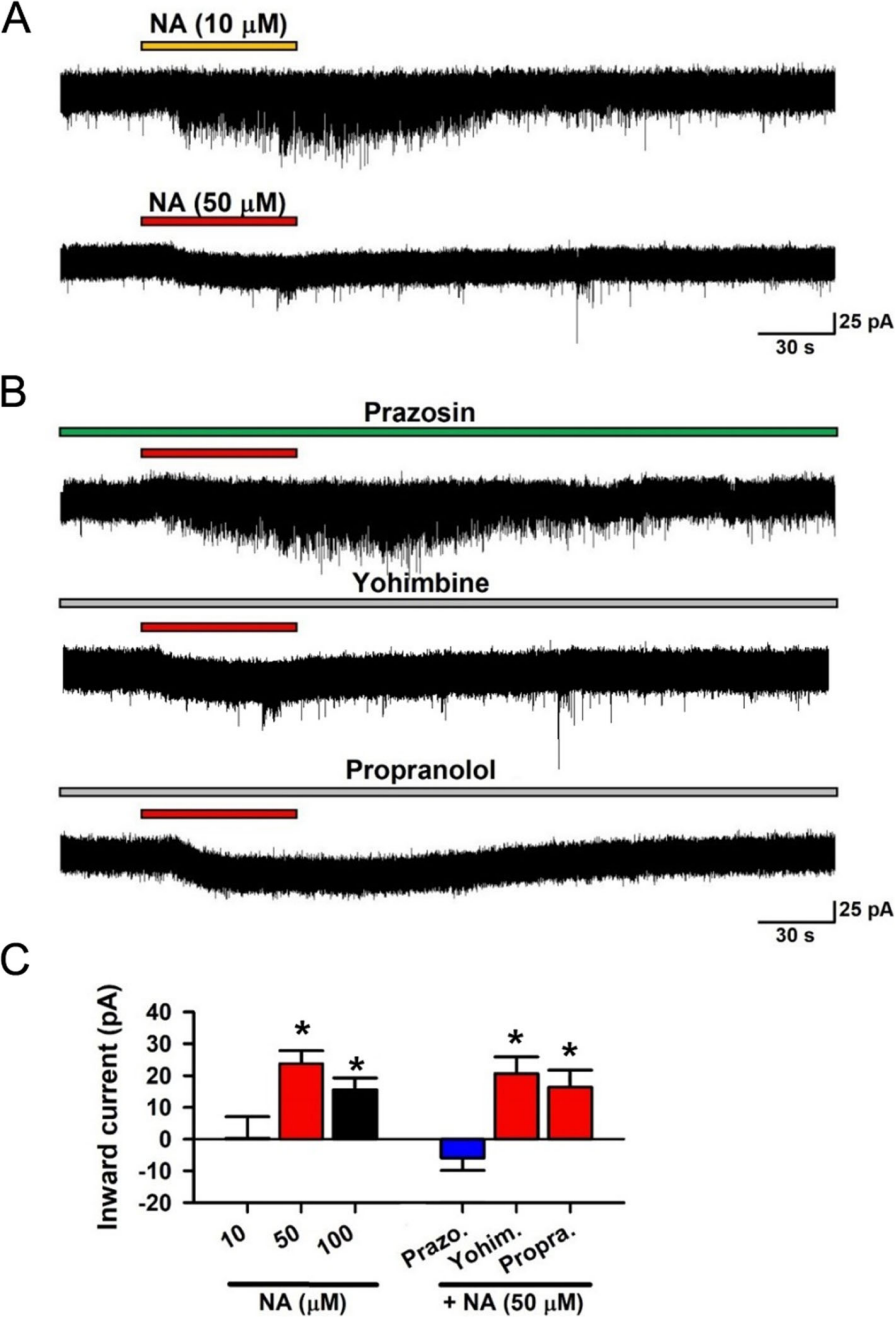
Noradrenaline induced inward current in pyramidal cells via α1 receptor. **a** Samples showing High dose (50 μM) but not low dose (10 μM) of NA produced inward currents. **b** Samples showing α1 receptors antagonist prazosin, but not α2 receptors antagonist yohimbine nor β1 receptors antagonist propranolol blocked the inward current induced by NA (50 μM). ***C,*** Averaged results showing high dose but not low dose of NA induced inward current (10 μM NA: *n* = 13, 50 μM NA: *n* = 12, 100 μM NA: n = 12). The inward currents were blocked by α1 receptors antagonist, but not α2 receptors nor β receptors antagonist. High dose of NA (50 μM) produced inward current is blocked by Prazosin (10 μM NA: 0.34 ± 3.74 pA, n = 13; 50 μM NA: 23.73 ± 4.13 pA, n = 12; 100 μM NA: 15.69 pA ± 3.82 pA, n = 12; Prazosin:: − 5.94 ± 3.76 pA, *n* = 9; Yohimbine: 20.58 ± 4.59 pA, *n* = 8; Propranolol: 16.35 ± 4.79 pA, n = 8). *P < 0.05, 10 μM NA vs. 50 μM or 100 μM NA, Prazosin vs. Yohimbine or Propranolol. One-Way ANOVA

Morphological evidence suggest that NA might regulate excitatory glutamatergic inputs, as well as the post-synaptic cellular activities, of pyramidal cells in the ACC. We next performed in vitro whole-cell patch-clamp recording from layer II/III pyramidal cells in the adult mice ACC to examine the modulatory effects of NA. Spontaneous excitatory postsynaptic currents (sEPSCs) were recorded. We found that NA (10–100 μM) enhanced the frequency of the sEPSC without affecting the amplitude (Fig. 2a-d). These effects were blocked by application of the β receptor antagonist propranolol (1 μM). While the α1 receptor antagonist prazosin (1 μM) or α2 receptor antagonist yohimbine (1 μM) had no effect on NA induced enhancement of sEPSC (Fig. 2e-f). In addition, high doses of NA (50 and 100 μM) also induced an inward current. This inward current was blocked by prazosin, but not by yohimbine or propranolol (Fig. 3). To test if the effects of NA might be selective for excitatory transmission in the ACC, we examined inhibitory transmission as well. We found that neither the frequency nor the amplitude of the spontaneous inhibitory postsynaptic currents (sIPSCs), recorded from the pyramidal neurons, were changed by NA (10 μM) application (Fig. 2g-h). This finding is in consistent with our morphological results and indicate that NA facilitates the presynaptic glutamate release to the pyramidal cells and enhances pyramidal cell membrane excitability, through distinct subtype receptors (β and α1 receptors, respectively).

**Fig. 4:**
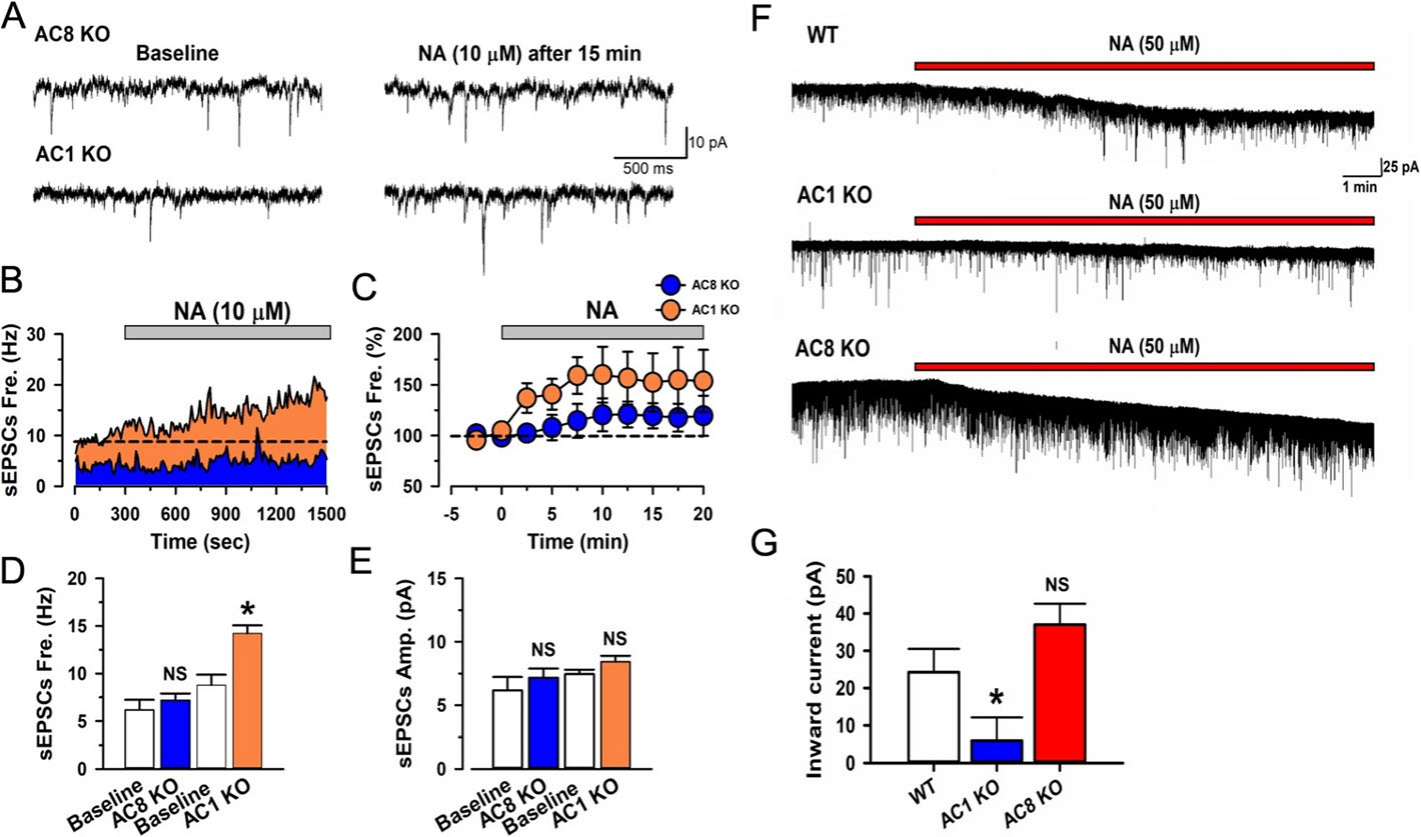
NA induced enhancement of glutamatergic transmission is blocked in different type of adenylyl cyclase knockout mice. **a** Sample traces of sEPSCs before and after the application of NA (10 μM in 15 min) in AC8 KO or AC1 KO mice. **b** Time course of sEPSCs for NA (10 μM) application in sample neurons of an AC8 or AC1 KO mice. **c** Averaged time course of sEPSCs for NA application from AC8 KO or AC1 KO mice. **d** Averaged frequency of sEPSCs with application of NA in AC8 KO or AC1 KO mice (Baseline in AC8 KO mice: 6.17 ± 1.05 Hz; AC8 KO mice: 7.17 ± 0.73 Hz, *n* = 7; Baseline in AC1 KO mice: 8.79 ± 1.08 Hz; AC1 KO mice: 14.22 ± 0.85 Hz, *n* = 6.). NS indicates no statistical significance between Baseline and AC8 KO mice. *P < 0.05, baseline vs. AC1 KO. Paired *t*-test. **e** Averaged amplitude of sEPSCs with application of NA in AC8 KO or AC1 KO mice (Baseline in AC8 KO mice: 7.75 ± 0.11 pA; AC8 KO mice: 8.21 ± 0.47 pA, n = 7; Baseline in AC1 KO mice: 7.46 ± 0.35 pA; AC1 KO mice; 8.43 ± 0.46 pA, n = 6). NS indicates no statistical significance between Baseline and AC8 KO mice or AC1 KO mice. Paired *t*-test. **f** Sample traces showing NA could induce inward currents in WT and AC8 KO mice but not in AC1 KO mice. ***G,*** The summarized data showing NA induced inward currents in WT or AC8 KO but not AC1 KO mice (WT: 21.28 ± 6.23 pA, *n* = 16; AC1 KO mice: 6.01 ± 6.13 pA, *n* = 8, AC8 KO mice: 37.013 ± 5.63 pA, n = 8). *P < 0.05, AC1 KO vs. WT and AC1 vs. AC8 KO mice. NS indicates no statistical significance between Baseline and AC8 KO mice. One-Way ANOVA

The β receptors and α1 receptors belong to members of the G protein-coupled receptor family (GPCR) and are associated with Gs and Gq, respectively [45]. Since adenylyl cyclases (ACs) are important downstream molecules for Gs activation and closely associated with Gq activation induced calcium influx [32, 46], we tested whether NA-enhanced excitatory transmission would be affected in mice with gene deletion of AC1 or AC8 (AC1 and AC8 KO mice), which are the two major neuronal and calcium-stimulated subtypes of ACs [34, 47] (Fig. 4). Interestingly, the enhanced sEPSCs by NA (10 μM) application was blocked in AC8 KO but not in AC1 KO mice (Fig. 4a-e). While the inward currents induced by application of 50 μM of NA was significantly reduced in AC1 KO but not in AC8 KO mice (Fig. 4f-g). These results suggest that AC8 and AC1 contribute to pre- and post-synaptic downstream signalling for NA’s effect on the excitatory transmission in ACC, respectively.

### In vivo study for NA’s enhancement of ACC neuronal activities

**Fig. 5:**
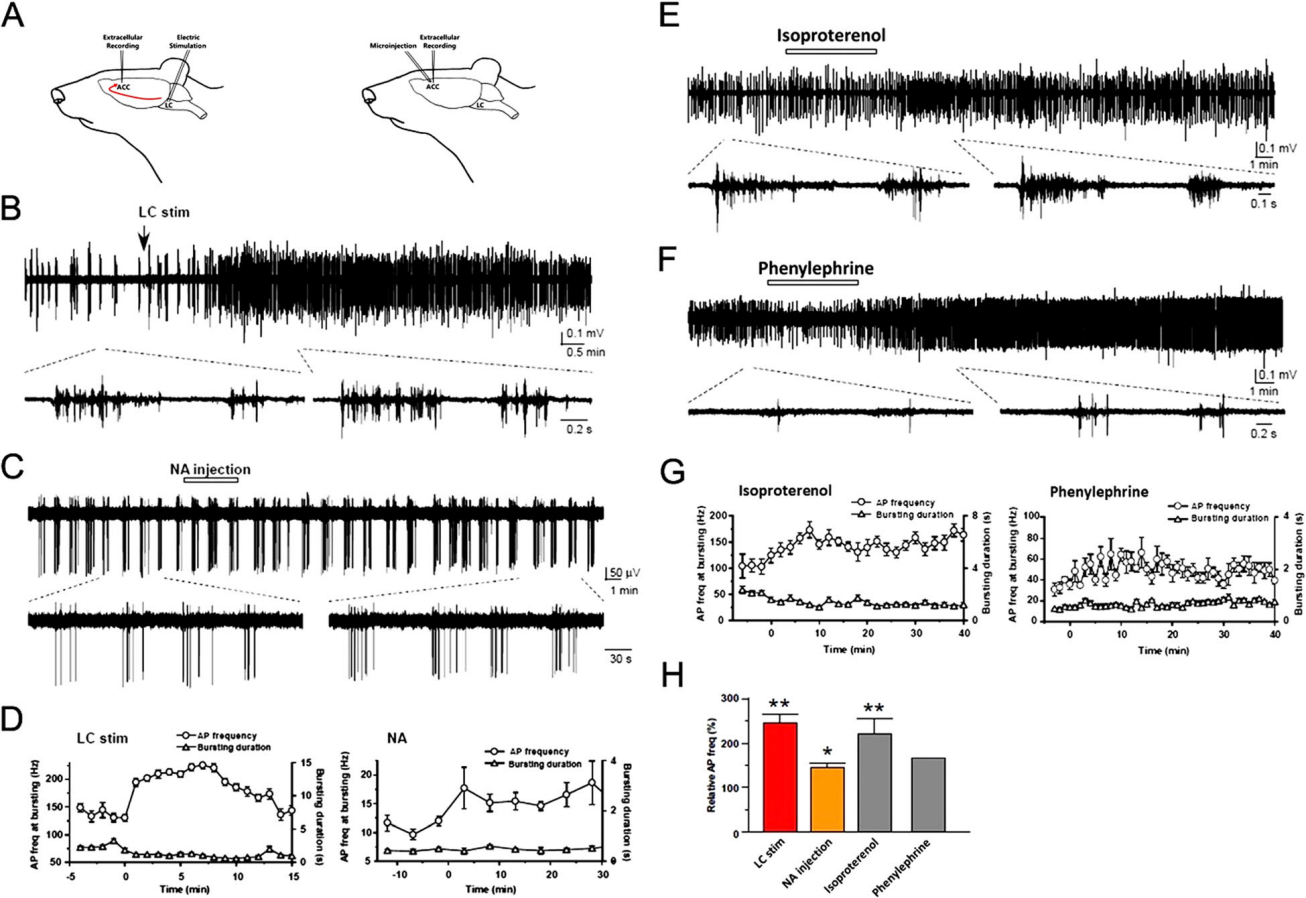
Activation of LC or application of NA enhances neuronal activities in the ACC in vivo*.***a** Scheme showing the extracellular recording from ACC neurons with electric stimulation in the LC or with the microinjection of NA into the ACC. **a** Sample trace showing electric stimulation of LC enhances the frequency of spikes in the ACC. **a** Sample trace showing microinjection of NA into the ACC increases the frequency of spikes in the ACC neurons. **d** Time course of spikes and bursting duration of the ACC neurons after electric stimulation of the LC in **b** or microinjection of NA in **c**. **e-f**, Sample traces showing local application of a β receptor agonist Isoproterenol (37 μg/0.5 μL) or α1 receptor agonist Phenylephrine (5 μg/0.5 μL) increased the frequency of spikes in the ACC. **g** Time course of spikes and bursting duration induced by microinjection of Isoproterenol (left) or Phenylephrine (right). **h** Relative frequency of spikes of ACC neurons induced by LC electric stimulation or microinjection into ACC with NA, Isoproterenol or Phenylephrine (LC stimulation: 226 ± 23% of baseline, *n* = 7; NA injection; 143 ± 12% of baseline, n = 5; Isoproterenol: 220 ± 33% of baseline, n = 12). *P < 0.05; **P < 0.01, compared with baseline. Paired *t*-test

In vitro brain slice experiments indicate that NA enhances excitatory transmission in the ACC. Next, we wanted to know if focal electrical stimulation of the LC or NA microinjection into the ACC may enhance the neuronal activity in the ACC under in vivo condition (Fig. 5a). Spontaneous bursting activities were detected in the ACC neurons from anaesthetized rats. The firings from pyramidal neurons in the ACC were identified with the spike width, as we reported previously [40]. We found that electrical stimulation of the LC increased the number of spikes in the ACC (Fig. 5b, d and h). Similar results were found with NA (50 μg/0.5 μL) microinjection into the ACC (Fig. 5c, d and h). Because β and α1 receptors are critical for the ACC excitation in vitro, we injected β receptor agonist Isoproterenol (37 μg/0.5 μL) or α1 receptor agonist Phenylephrine (5 μg/0.5 μL) into the ACC. We found that microinjection of the agonists enhanced the firing frequency during bursting activities (Fig. 5e-h).

### Optostimulation of LC-ACC pathways

**Fig. 6:**
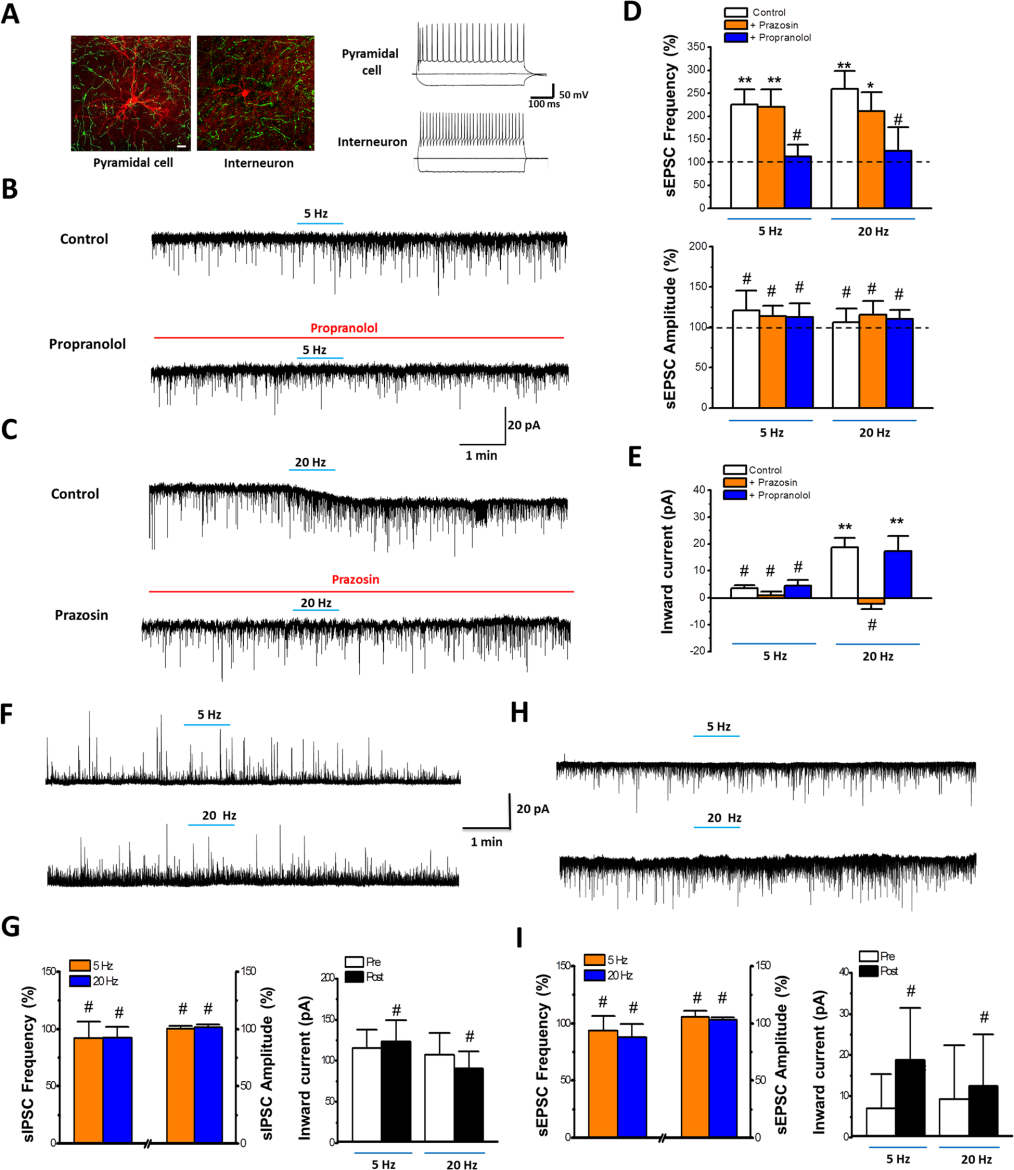
Optostimulation of LC-ACC projecting fibres facilitates glutamatergic synaptic transmission onto pyramidal cells in the ACC. **a** Samples showing the morphological and electrophysiological characteristics of a patched pyramidal cell and interneuron (red) in layer III of the ACC, as well as the NAergic projecting fibers from the LC (green), which are optostimulated by 470 nm blue light. Bar equals to 10 μm. **b** Sample traces showing that the increased frequency of sEPSC from pyramidal cells, by exposure to low frequency (5 Hz) optostimulation, is blocked by β receptor antagonist Propranolol. **c** Samples traces showing that high frequency (20 Hz) optostimulation induced inward current is blocked by α1 receptor antagonist Prazosin. **d** (Upper panel) Averaged results showing 5 or 20 Hz optostimulation increases the frequency of the sEPSCs (5 Hz, 225.8 ± 32.2% of baseline; 20 Hz, 259.4 ± 38.7% of baseline. *n* = 10 neurons in each group), which is blocked by β receptor antagonist Propranolol (5 Hz, 112.2 ± 26.2% of baseline; 20 Hz, 124.3 ± 51.7% of baseline. n = 8 in each group) but not by α1 receptor antagonist Prazosin (5 Hz, 219.9 ± 38.6% of baseline; 20 Hz, 211.1 ± 40.8% of baseline. n = 10 in each group). (Bottom panel) Averaged results showing that 5 or 20 Hz optostimulation do not change the amplitude of the sEPSCs (5 Hz, 120.8 ± 24.4% of baseline; 20 Hz, 106.3 ± 16.5% of baseline. n = 10 in each group), neither do the application of Prazosin (5 Hz, 113.7 ± 13.1% of baseline; 20 Hz, 115.3 ± 17.5% of baseline. n = 10 in each group) nor Propranolol (5 Hz, 112.3 ± 17.3% of baseline; 20 Hz, 110.2 ± 11.2% of baseline. n = 8 in each group). **e** Averaged results showing that 20 Hz but not 5 Hz (5 Hz, 3.58 ± 0.92 pA, 20 Hz, 18.7 ± 3.45 pA, n = 10 in each group) optostimulation induces inward currents, which is blocked by Prazosin (5 Hz, 0.88 ± 1.43 pA, 20 Hz, − 2.27 ± 1.93 pA, n = 10 in each group) but not by Propranolol (5 Hz, 4.41 ± 2.15 pA, 20 Hz, 17.3 ± 5.61 pA, n = 8 in each group). **f-g** Sample traces and averaged results showing that exposure to 5 or 20 Hz optostimulation changes neither the frequency or amplitude of the sIPSC (frequency: 5 Hz, 91.9 ± 14.3% of baseline, 20 Hz, 92.2 ± 9.7% of baseline; amplitude: 5 Hz, 100.2 ± 2.5% of baseline, 20 Hz, 101.6 ± 2.6% of baseline) nor the holding current (5 Hz: pre, 115.4 ± 22.4 pA, post, 123.5 ± 25.6 pA; 20 Hz, pre, 106.9 ± 26.5 pA, post, 90.3 ± 20.6 pA) of pyramidal cells. **h-i***,* Sample traces and averaged results showing that exposure to 5 or 20 Hz optostimulation changes neither the frequency or amplitude of the sEPSC (frequency: 5 Hz, 93.5 ± 12.8% of baseline, 20 Hz, 87.9 ± 11.6% of baseline; amplitude: 5 Hz, 105.6 ± 5.3% of baseline, 20 Hz, 103.2 ± 2.2% of baseline) nor the holding current (5 Hz: pre, 7.0 ± 8.3 pA, post, 18.8 ± 12.7 pA; 20 Hz, pre, 9.3 ± 13.1 pA, post, 12.4 ± 12.6 pA) of interneurons. #, *P* > 0.05; *, *P* < 0.05; **, *P* < 0.01; Each is compared with the baseline. Paired *t*-test

The NAergic effect on ACC neurons were then further confirmed by selectively activating the LC-ACC NAergic projection by Cre-dependent optostimulation. Bilateral LCs were infected by injecting AAV-DIO-ChR2(H134R)-eYFP in DBH-cre mice. The NA projecting fibers in the ACC were optostimulated by low (5 Hz) or high (20 Hz) frequency of blue light (470 nm) after ACC slices were prepared from infected mice (Fig. 6a). We found that both 5 and 20 Hz optostimulation for 1 min increased the frequency of the sEPSCs on the recorded pyramidal cells in the ACC, without changing the amplitude of sEPSCs (Fig. 6b-d). However, only 20 Hz but not 5 Hz optostimulation induced obvious inward current (Fig. 6b, c and e), similar to the requirement of high doses of NA for producing postsynaptic effects. Furthermore, the facilitation effect on the frequency of sEPSCs was totally blocked by propranolol and the induced inward current was blocked by prazosin. Consistent results from both rats and mice indicate the NA induced facilitation effect is unlikely specie dependent.

The sIPSCs of the pyramidal cells and sEPSCs of the interneurons were also recorded. However, 5 and 20 Hz optostimulation neither changed the frequency and amplitude of the sIPSCs or sEPSCs, nor induced inward or outward currents, of the pyramidal cells or interneurons (Fig. 6f-i). Considering that the sEPSCs were enhanced and the inward currents were induced in pyramidal cells, we proposed that the difference may be in part due to distinct distribution of the β and α1 receptors in pyramidal cells and interneurons.

### Stimulation of LC-ACC pathway induced behavioural responses

**Fig. 7:**
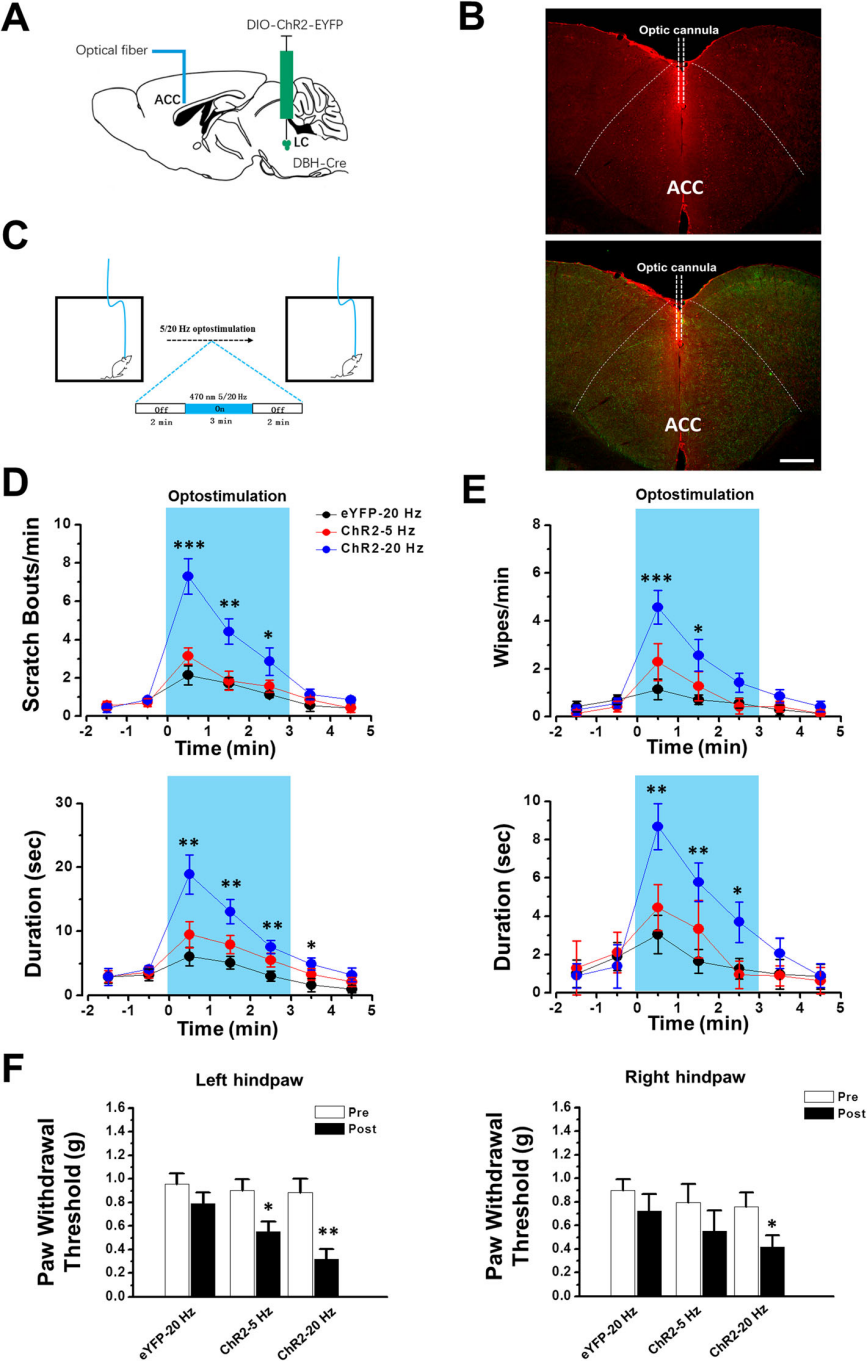
Activation of LC-ACC pathway facilitates the itch and pain sensations. **a** Diagram of stereotaxic virus injection into the LC of DBH-Cre mice and optic cannula implantation in the ACC. **b** Figures showing that optostimulation of the LC-ACC projecting NAergic fibers (Green) through the optic cannula induced strong Fos expression (Red) within the ACC. **c** Experimental schema of behaviour test. **d** The bouts and duration of scratching are increased after in vivo optostimulation in mice with ChR2 infection. * *P* < 0.05, **, *P* < 0.01, ***, *P* < 0.001, in comparison with eYFP-20 Hz group (n = 7 mice in each group). Two-Way ANOVA. **e** The number and duration of wiping are increased after in vivo optostimulation. * *P* < 0.05, **, *P* < 0.01,***, *P* < 0.001, in comparison with eYFP-20 Hz group. Two-Way ANOVA. **f** The paw withdrawal thresholds are decreased in both left and right hind paws after optostimulation (left hindpaw: eYFP-20 Hz: pre, 0.96 ± 0.09 g, post, 0.79 ± 0.09 g, *P* > 0.05; ChR2–5 Hz: pre, 0.90 ± 0.10 g, post, 0.55 ± 0.08 g, *P* < 0.05; ChR2–20 Hz: pre, 0.88 ± 0.12 g, post, 0.32 ± 0.09 g, *P* < 0.01; right hindpaw: eYFP-20 Hz: pre, 0.90 ± 0.09 g, post, 0.73 ± 0.14 g, *P* > 0.05; ChR2–5 Hz: pre, 0.80 ± 0.16 g, post, 0.55 ± 0.17, *P* > 0.05; ChR2–20 Hz: pre, 0.76 ± 0.12 g; post, 0.42 ± 0.10 g, *P* < 0.05. *n* = 6 mice in each group). Paired *t*-test

Our in vivo and in vitro results consistently indicate that NAergic LC-ACC projection potentiatesexcitatory synaptic transmission in the ACC. Considering the importance of ACC neurons in the sensory process of pain and itch, we liked to examine the behavioral effects after optostimulating the NAergic LC-ACC projection in vivo. In DBH-Cre mice with ChR2 infection of bilateral LCs, optic cannula was implanted in the ACC and the animals basal behaviors were evaluated after 470 nm optostimulation were applied (Fig. 7a) [15]. The distribution of Fos protein in the ACC strongly suggest that opto-stimulation is limited within ACC (Fig. 7b). We found that, after the optostimulation, both scratching with the hind paws and wiping with the fore paws on animal’s face and head were observed (Fig. 7c-d). The scratching behavior has been proposed to link to itching responses and wiping behavior has linked to nociceptive responses, respectively [48]. We further tested the paw withdrawal threshold by von Frey filament stimulation on animals’ hind paws. The mechanical thresholds were significantly decreased after 20 Hz optostimulation (Fig. 7e). On the contrary, in control mice with AAV-DIO-eYFP injection, 20 Hz optostimulation did not produce similar effects (Fig. 7c-e).

## Discussion

Noradrenergic projections from the LC to the CNS have been implicated in many key brain functions, such as attention, arousal, learning and memory [1, 2, 49, 50]. Our present studies reveal a novel function of NAergic projection from LC to ACC, an important cortical region for the regulation of sensory process [20, 22, 23, 51]. On the contrary to the well-known inhibitory effect of sensory transmission at the spinal level, NA’s effect on ACC seems to facilitate the sensory process. In the situation encounters noxious stimuli (that causes pain sensation), activation of NAergic LC-spinal pathway will reduce the amount of nociceptive inputs to the spinal cord [17–19] but enhance the nociceptive responses in the brain.

The LC sends descending NAergic projections to the spinal cord [52], which is a major inhibitory pathway and alleviates chronic pain in the spinal cord [6, 13, 14]. This inhibitory effect is mainly mediated by α_2A_ receptors, which are located on afferent axon inputs in the superficial laminae [53], and α_2C_ receptors, which are located on glutamatergic interneurons, in spinal dorsal horn [54]. Meanwhile, NA enhances neural excitability in GAD67 expressing interneurons in the laminae II through activation of α1 receptor [16]. One in vivo electrophysiological experiment provides direct evidence showing the inhibitory effect of NA in spinal level, in which optogenetic stimulation of the LC-spinal cord pathway facilitates inhibitory transmissions in the superficial dorsal horn neurons [8]. This inhibition is likely mediated by both reducing the excitatory nociceptive inputs and activation of local inhibitory interneurons.

Different from the well-known descending inhibition for nociception, the effect of LC-cortical ascending pathway in pain sensation is not clear yet. Recently, Hirschberg et al., show that activation of LC-prefrontal cortex (PFC) induces aversion and anxiety and exacerbated spontaneous pain in neuropathic pain rats. However, the mechanism for the LC-PFC ascending regulation is not investigated [55]. In the present study, we found that NA from LC-ACC enhanced the excitatory transmission in the ACC, through α1 and β receptors. Light and electron microscopic observation combined with Cre-dependent tracing method provided morphological evidence that the ascending NAergic projections from LC predominately terminated on the pyramidal neurons but not interneurons in the ACC. Functionally, bath application of NA enhanced presynaptic glutamatergic synaptic transmission to and postsynaptic cellular excitability of layer II/III pyramidal neurons, through β and α1 receptors respectively. This is confirmed by optogenetic stimulation of LC-ACC projections. Additional in vivo unit recordings confirmed that electrical stimulating of the LC or local application of NA enhanced neural activity in the ACC. Therefore, the different mechanisms between descending inhibition and ascending facilitation pathway from the LC may be due to the different types of NAergic receptors and target neurons in the spinal cord and the cortex.

An interesting role of NA in the ACC was that NA played different effects in a dose-dependent manner. Low dose of NA or low frequency optostimulation to LC-ACC fibers strongly increased the release of glutamate to layer II/III pyramidal cells in the ACC. On the other hand, high dose of NA or high frequency optostimulation, in addition to increased glutamate release, induced inward current in recorded pyramidal cells. It’s also shown that the increased glutamate release was mediated by β receptor and the induced inward current was mediated by α1 receptor. Using AC1 or AC8 KO mice, we confirmed that the enhanced release of glutamate was blocked in AC8 KO mice and the induced inward current was blocked in AC1 KO mice. Thus, the facilitatory effect produced by NA in ACC may be mediated by presynaptic β receptor-AC8 signaling pathway and post-synaptic α1 receptor-AC1 signaling pathway, respectively. This finding is in consistent with our previous works, in which we have confirmed that AC-cAMP signaling pathways are involved in different forms of pain in the ACC [24, 34, 56, 57]. In the present study, except for the mechanical hypersensitivity effect, we found that the scratching behavior were enhanced after activating the NAergic projection fibers. Thus, activations of AC-cAMP signaling pathway may not be limited for the regulation of nociceptive information. It is more likely that the pathway is involved with the hypersensitivity for both pain and itching-like sensory information.

It is known that the ACC plays critical roles in both pain and itch in from rodents to human [28, 30, 51]. In animal study, nociceptive stimulation at the hindpaw increases neural activity in the ACC in vivo [58]. Animal models of chronic pain enhance glutamatergic transmission within the ACC [51, 59, 60]. In addition, itch stimulation also increases glutamatergic transmissions in the ACC [30, 61]. Interestingly, activations of pyramidal neurons by optogenetic stimulations within the ACC enhance both pain and itch behaviors [26, 31]. However, it is unclear whether pain and itch information share the common pathway in the CNS under physiological and pathophysiologic conditions. How does activation of NA from the LC in the ACC regulate pain and itch functions is not investigated before. However, according to our results, NA may modulate both pain and itch pathway within the ACC by altering glutamatergic transmissions. Further study is certainly needed for understanding the mechanism of how NA modulate these two different sensations in detail.

In sum, we find that NAergic LC-ACC projection facilitates the excitatory synaptic transmission to pyramidal cells in the ACC and enhance the itch and pain like responses in animals. This LC-ACC ascending projection may help animals or humans to enhance behavioral responses to injury, and alert themselves from dangerous situation. It may also help to form long-term memory at cortical synapses [50], which is beneficial for human or animals to gain new knowledge from potentially dangerous information.

## Data Availability

The data that support the findings of this study are available from the corresponding author upon reasonable request.

## Abbreviations

AC: Adenylyl cyclase
ACC: Anterior cingulate cortex
ACSF: Artificial cerebrospinal fluid
CNS: Central nervous system
IC: Insular cortex
KO: Knockout
LC: Locus coeruleus
NA: Noradrenaline
PFC: Prefrontal cortex
sEPSCs: Spontaneous excitatory post-synaptic currents
sIPSCs: Spontaneous inhibitory postsynaptic currents
