# Correction to: Ascending noradrenergic excitation from the locus coeruleus to the anterior cingulate cortex

**DOI:** 10.1186/s13041-020-00692-4

**Published:** 2020-11-13

**Authors:** Kohei Koga, Akihiro Yamada, Qian Song, Xu-Hui Li, Qi-Yu Chen, Ren-Hao Liu, Jun Ge, Cheng Zhan, Hidemasa Furue, Min Zhuo, Tao Chen

**Affiliations:** 1 Center for Neuron and Disease, Frontier Institute of Science and Technology, Xi’an Jiaotong University, Xi’an, 710049 China; 2 Department of Physiology, Faculty of Medicine, University of Toronto, Medical Science Building, 1 King’s College Circle, Toronto, ON M5S 1A8 Canada; 3 Department of Neurophysiology, Hyogo College of Medicine, Nishinomiya, 663-8501 Japan; 4 Department of Anatomy, Histology & Embryology, Air Force Medical University, Xi’an, 710032 China; 5 National Institute of Biological Sciences, Beijing, 102206 China; 6 Tsinghua Institute of Multidisciplinary Biomedical Research, Tsinghua University, Beijing, 102206 China

## Correction to: Molecular Brain (2020) 13:49 10.1186/s13041-020-00586-5

Following publication of the original article [1], the authors identified an error in Fig. 3: repeated figures were used in Fig. 3a (lower panel) and Fig. 3b (middle panel). To correct this, the original Fig. 3a (lower panel) was replaced with a new sample figure. The correct complete Fig. 3 and its caption are given below and the original article has been corrected.

**Fig. 3:**
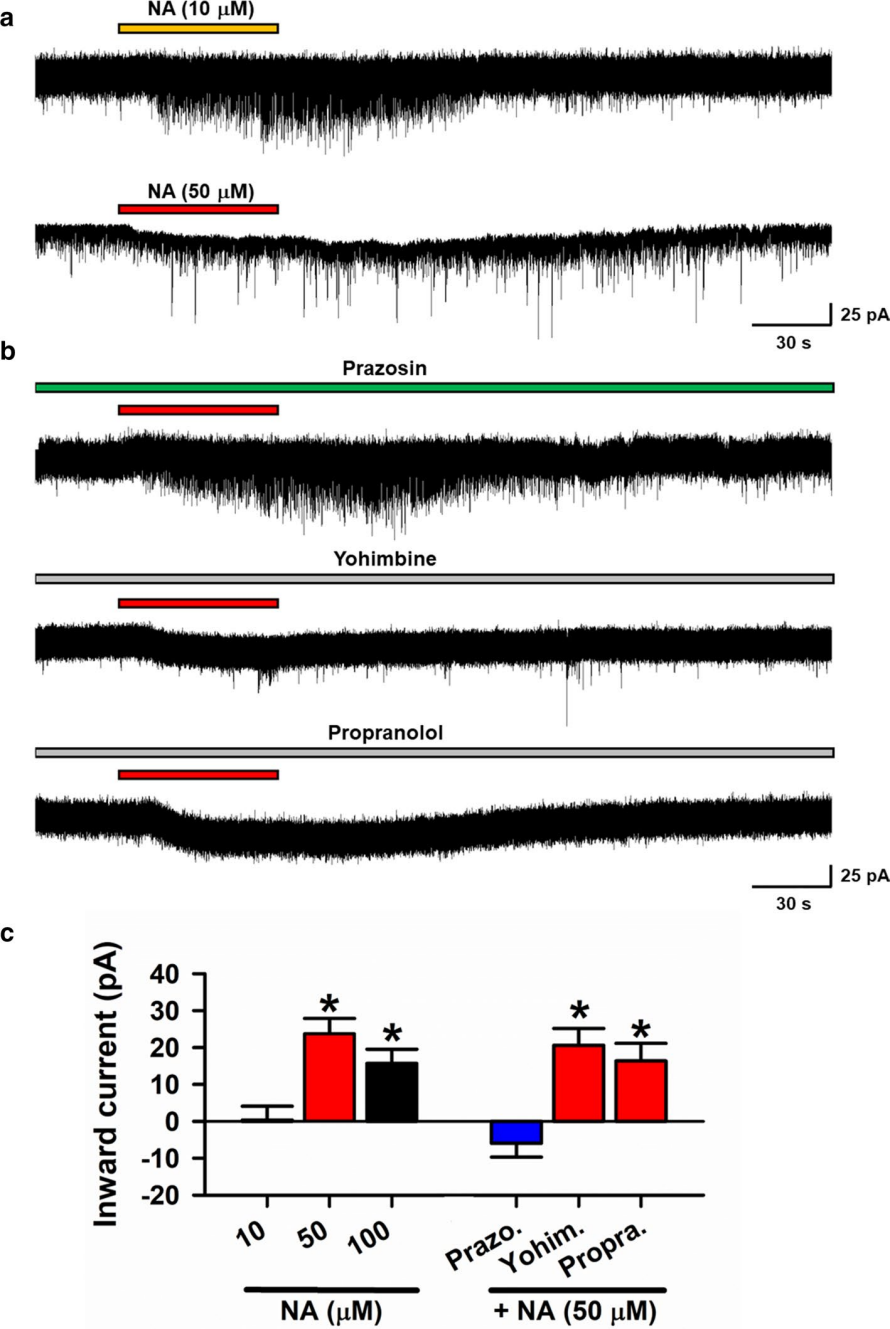
Noradrenaline induced inward current in pyramidal cells via α1 receptor. **a** Samples showing High dose (50 μM) but not low dose (10 μM) of NA produced inward currents. **b** Samples showing α1 receptors antagonist prazosin, but not α2 receptors antagonist yohimbine nor β1 receptors antagonist propranolol blocked the inward current induced by NA (50 μM). **c** Averaged results showing high dose but not low dose of NA induced inward current (10 μM NA: n = 13, 50 μM NA: n = 12, 100 μM NA: n = 12). The inward currents were blocked by α1 receptors antagonist, but not α2 receptors nor β receptors antagonist. High dose of NA (50 μM) produced inward current is blocked by Prazosin (10 μM NA: 0.34 ± 3.74 pA, n = 13; 50 μM NA: 23.73 ± 4.13 pA, n = 12; 100 μM NA: 15.69 pA ± 3.82 pA, n = 12; Prazosin:: − 5.94 ± 3.76 pA, n = 9; Yohimbine: 20.58 ± 4.59 pA, n = 8; Propranolol: 16.35 ± 4.79 pA, n = 8). *P < 0.05, 10 μM NA vs. 50 μM or 100 μM NA, Prazosin vs. Yohimbine or Propranolol. One-Way ANOVA
